# Understanding the Influences of EEG Reference: A Large-Scale Brain Network Perspective

**DOI:** 10.3389/fnins.2017.00205

**Published:** 2017-04-13

**Authors:** Xu Lei, Keren Liao

**Affiliations:** ^1^Sleep and NeuroImaging Center, Faculty of Psychology, Southwest UniversityChongqing, China; ^2^Key Laboratory of Cognition and Personality of Ministry of EducationChongqing, China

**Keywords:** EEG reference, large-scale networks, average reference, reference electrode standardization technique

## Abstract

The influence of reference is a critical issue for the electroencephalography (EEG) and event-related potentials (ERPs) studies. However, previous investigations concentrated less on the location of source at a systematic neuroscience level. Our goal was to examine the EEG signal associated with the locations from a common network parcellation of the human brain function, offering a system perspective of the influence of EEG reference. In our simulation, vertices uniformly distributed in eight large-scale brain networks were adopted to generate the scalp EEG. The brain networks contain the visual, somatomotor, dorsal attention, ventral attention, limbic, frontoparietal, default networks, and the deep brain structure. The distributions of the most sensitive and neutral electrodes were calculated for each network based on the lead-field matrix. While the most sensitive electrode had a network-specific symmetric pattern, the electrodes in scalp surface had approximately equal chance to be the most neutral electrode. Simulated data were referenced at the FCz, the Oz, the mean mastoids (MM), the average (AVE), and the infinity reference obtained by the reference electrode standardization technique (REST). Intriguingly, the relative error followed the pattern REST<AVE<MM<(FCz, Oz), regardless of the number of electrodes and signal-to-noise ratios. Our findings suggested that REST was a potentially preferable reference for all large-scale networks and AVE virtually performed as REST under several conditions. As EEG and ERPs experiments within the same behavioral domain always have activations in some specific brain networks, the comparisons revealed here may provide a valuable recommendation for reference selection in clinical and basic researches.

## Introduction

Electroencephalography (EEG) is a real-time, noninvasive measure of neuronal activity, which is considered as a valuable and cost-effective tool for the study of brain function in a wide range of clinical and basic research. The recent developments of EEG have allowed an increased topographic accuracy with high-density montage systems, the improved data quality with hardware updating and the reduced preparation time with dry electrode (Kleffner-Canucci et al., 2012; Mullen et al., 2015). Additionally, the opportunities to combine scalp EEG with other imaging modalities, as well as with robotics or neurostimulation, have made this technique more attractive for many emerging research fields.

However, an unsolved critical issue for the EEG studies is the choice of the EEG reference. In fact, both the evoked and spontaneous potentials of neural activities are influenced by reference, because each EEG electrode only yields information about the difference of electrical activity between two positions on the head (Nunez, 1981; Hagemann et al., 2001; Yao, 2001). It is indispensable to set a physical reference during EEG recordings and the signal in each electrode is obtained as the difference between the electric potentials in its location and in the location of the reference electrode. Here, the physical references used during recording is different from the computational references used during re-reference. The former includes the FCz, Oz, linked ears, nose, and neck ring, while the latter includes the mean mastoids, the average and the reference electrode standardization technique (REST) (Yao, 2001). If the neural electric potential near the reference electrode is not neutral, the measurement will be contaminated inevitably at all the other electrode sites, and further distorts the temporal dynamic analysis and power spectra analysis of the EEG recording. As a result, the preferential use of different reference schemes has evolved for individual research teams and led to de facto conventions for specific research fields or clinical practice (Kayser and Tenke, 2010).

Currently, there is no universally accepted reference scheme, hindering across-study comparability (Kayser and Tenke, 2010; Nunez, 2010). One commonly applied reference is the mean mastoids, which assumed the sites around the mastoids are free from activity of neural source. This assumption is always violated, because there is no single location where the potential can be considered to be completely neutral. The average reference has obtained large consensus thanks to its assumption of that the surface integral of the electric potential over a volume conductor containing all the current sources is zero (Bertrand et al., 1985). As the number of electrodes is increased and the coverage of the whole brain is approachable, it is increasingly believed that the average potential over all the electrodes provides a virtual zero-potential point. An alternative approach latter proposed by Yao was REST (Yao, 2001). REST transforms the EEG potentials referenced at any scalp points into the potentials referenced to a point located at infinity, far from all the possible neuronal sources and thus acting as an ideal neutral reference location. The merit of REST has been proved in event-related potentials (ERPs) (Tian and Yao, 2013), EEG spectrum (Yao et al., 2005), EEG coherence (Marzetti et al., 2007), and network analysis (Qin et al., 2010; Chella et al., 2016).

Previous investigations are confined to associating the selection of reference with some experiment paradigms (Hagemann et al., 2001) or source configurations (Marzetti et al., 2007), and rare has investigated the impact of the localization of EEG source on reference selection, due to lack of large-scale brain functional network templates. Fortunately, Yeo et al. (2011) adopted a data-driven clustering approach using 1,000 resting-state fMRI studies and segmented seven cortical neuronal networks from the cerebral cortex: the visual, somatomotor, dorsal attention, ventral attention, limbic, frontopariental, and default mode networks. Based on the general assumption that EEG and ERPs experiments on the same psychological process normally activates certain brain networks, comparing the effects of different references within a template of brain network may unravel the potentially effective choice of reference in each behavioral domain. Our goal was to examine the EEG signal associated with the locations from a common network parcellation of the human brain function, offering a systems-neuroscience perspective of effect of the EEG reference.

We utilized a high-density canonical cortical mesh with 8,196 vertices to simulate the scalp EEG signal and each vertex was uniformly distributed in eight large-scale brain networks. The simulated signal was referenced to FCz, Oz, the mean mastoids (MM), the common average (AVE) of all electrodes and the infinity reference recovered by the Reference Electrode Standardization Technique (REST). The number of electrodes with 32, 64, and 128 and the signal-to-noise ratios with 2, 4, 8, 16, and 32 were included as influencing factors for the effect of reference electrode. Our systematic neuroscience comparisons among large-scale brain networks may provide valuable recommendations of reference selection for EEG and ERP studies.

## Methods

The influence of reference electrodes (FCz, Oz, MM, AVE) and the infinity reference recovered by the REST method were investigated through the simulated EEG potentials under different densities of electrode and signal-to-noise ratios. The spatial distribution and the time course for each large-scale brain functional network are described in the following.

### Spatial distribution of EEG source and the head model

**Figure 1:**
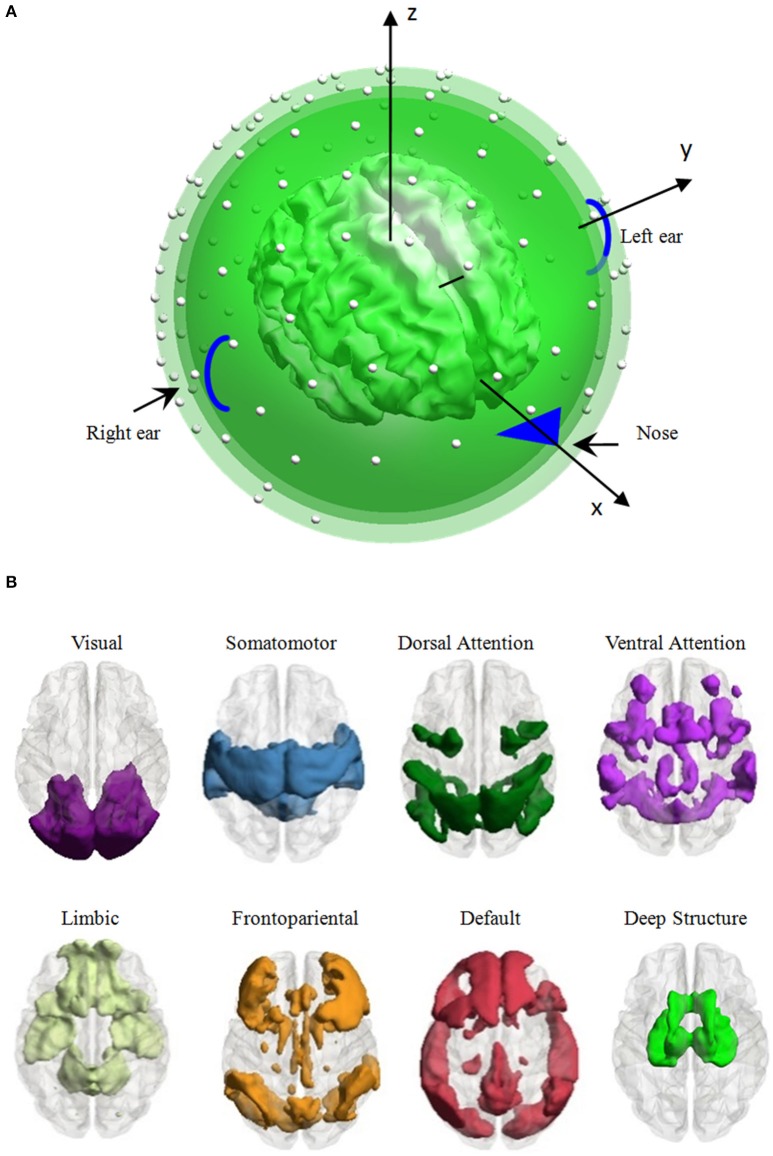
**Head model and the distribution of eight large-scale brain functional networks. (A)** Head model: a high-density canonical cortical mesh (inner irregular object) with 8,196 vertices within a concentric three-sphere head model, with electrodes (the white dots) on the upper surface. **(B)** Vertex, which was used to generate EEG signal per simulation, is distributed in one of the eight large-scale brain networks, i.e., the visual, somatomotor, dorsal attention, ventral attention, limbic, frontoparietal, default networks, and the deep brain structure.

The EEG forward model is restricted to a high-density canonical cortical mesh (Figure 1). The mesh has 8,196 vertices and was extracted from a structural MRI of a neurotypical male in Fieldtrip software (http://fieldtrip.fcdonders.nl/download.php). Vertices were uniformly distributed on the gray-white matter interface, and assumed as potential dipoles oriented perpendicular to the surface. The electrodes of EEG system were registered to the scalp surface, and the lead-field matrix was calculated analytically using a three-shell spherical head model including scalp, skull, and brain (de Munck, 1988). As the EEG forward problems was solved by using the analytic expansion, an implicit assumption is that the lead field is based on the reference at infinity. The normalized radii of the three-shell spheres were 0.87 (inner radius of the skull), 0.92 (outer radius of the skull) and 1.0 (radius of the scalp). The normalized conductivities were 1.0, 0.0125, and 1.0 for the brain, skull and scalp, respectively. For the number of electrodes *n*, the lead-field ***L*** was a matrix with dimension *n* × 8,196.

Each vertex was adopted to generate the scalp EEG recording per simulation. As the vertex belongs to one of the eight large-scale brain networks, we averaged all the performance of the vertices belonging to a network to evaluate the influence of the network parcellation on the EEG data. Seven large-scale networks were identified based on 1000 resting-state functional connectivity, including the visual, somatomotor, dorsal attention, ventral attention, limbic, frontoparietal, and default networks (Yeo et al., 2011). Considering the importance of the deep brain structure (thalamus, caudate, hippocampus, amygdala and olfactory), we used the automated anatomical labeling (AAL) parcellation atlas (Tzourio-Mazoyer et al., 2002) to construct an eighth large-scale network comprising these structures. The 8,196 vertices were separated to eight subsets depended on its nearest neighbor voxel in the large-scale brain network templates. The number of vertices of each network ranged from 428 to 1,572 (1,255 vertices in the visual network, 1,539 vertices in the somatomotor network, 979 vertices in the dorsal attention network, 842 vertices in the ventral attention network, 513 vertices in the limbic network, 1,068 vertices in the frontoparietal network, 1,572 vertices in the default network, and 428 vertices in the deep brain structure, see Figure 1B for the spatial pattern of each network).

### Signal strength and the sensitive/neutral electrode

As the lead-field matrix ***L*** was calculated for each dipole with a unit strength, the *i*th column ***L***_*i*_ (corresponding to the *i*th dipole and *i* = 1, …, 8,196) is a measurement for how strong the signal of the source can be observed from every electrode on the scalp surface. The relative strengths in ***L***_*i*_ have direct relation with the sensitivity of electrodes (Rush and Driscoll, 1969). For example, if the absolute value of ***L***_*im*_ (the *m*th row in ***L***_*i*_) is larger than the absolute value of ***L***_*in*_ (the *n*th row in ***L***_*i*_), it means the *m*th electrode is more sensitive than the *n*th electrode. The reciprocity theorem shows that the sensitivity of the electrode is maximum to the dipolar sources oriented in the direction of the lead-field (perpendicular to the lines), and falls off as the cosine of the angle between the source and lead-field directions (Rush and Driscoll, 1969). We defined the electrodes corresponding to the maximum and minimum absolute values in ***L***_*i*_ as the most sensitive and neutral electrodes, respectively. We calculated the global sensitivity of the *i*th dipole to be reflected in the scalp as:

(1) $$\begin{array}{r} S_{i}=\frac{1}{\sqrt{n}}∥L_{i}∥ \end{array}$$

where ||***L***_*i*_|| is the Frobenius norm of the lead-field column corresponding to the *i*th dipole and *n* is the number of electrodes. We will use the averaged of the global sensitivity in the *k*th network for comparison among the montages with different number of electrodes.

Because 64-electrode is widely applied in experimental study, we focused our analysis on the electrode level with the lead-field matrix of 64-electrode. As the maximum or minimum absolute value in ***L***_*i*_ corresponds to the most sensitive or neutral electrode to reflect the source activity, we counted the times of electrode *j* that was selected as the most sensitive or neutral electrode for network *k*.

(2) $${\text{S}}_{\text{ k}}^{\text{ j}}=\frac{1}{m_{k}}\;\sum_{\left(i\;\in \;network\;k\right)}b_{i}$$

(3) $${\text{N}}_{\text{ k}}^{\text{ j}}=\frac{1}{m_{k}}\;\sum_{\left(i\;\in \;network\;k\right)}w_{i}$$

where *b*_*i*_ = 1 (*w*_*i*_ = 1) if the *j*th row in vector ***L***_*i*_ has the maximum (minimum) absolute value, otherwise *b*_*i*_ = 0 (*w*_*i*_ = 0). *m*_*k*_ is the number of vertices in brain network *k*. Notice that ${\text{S}}_{k}^{j}$ or ${\text{N}}_{k}^{j}$ would be equal to 1/64 if all the electrodes have the same chance to be the most sensitive or neutral electrode.

### Simulation description

We simulated the temporal process of a dipolar neural source by employing a damped Gaussian function:

(4) $$\begin{array}{rcl} {\text{y}}_{\text{k}}\; & = & \text{ exp}\left(-2\text{πτ }\times \text{ k}\times \text{ dt}\right)\\ \times \text{ cos}\left(2\pi f\times \text{ k }\times \text{ dt}\right),\text{k}=1,\ldots ,\;500 \end{array}$$

where dt = 2 ms, τ = 2 Hz, and *f* = 10 Hz. We chose this function because it looks like an evoked potential and was utilized in previous simulations (Yao, 2001). Using the function y and the lead-field ***L***, we derived the spatiotemporal recordings of *i*th vertex (*i* = 1, …, 8,196 and each simulation used one vertex to generate signal): ***V***_sim_ = ***L***_*i*_y with size *n* × 500, where ***L***_*i*_ is the *i*th column of ***L***.

We examined the effect of the number of electrodes *n* by comparing the performance of electrode configuration with 32, 64, and 128 electrodes. All electrode configurations were down-sampled from the standard 10-5 system (Oostenveld and Praamstra, 2001) to obtain an approximate uniform sample of the upper head. For each configuration, FCz, Oz, TP9, and TP10 were included, in order to simulate the reference electrodes. TP9 and TP10 were averaged to construct the mean mastoids (MM) reference. The effects of Gaussian white noise Σ was investigated by varying the signal-to-noise ratio with values of 2, 4, 8, 16, and 32, which were calculated as the ratio between the mean variance across channels of the signal ***V***_sim_ and the variance of noise Σ. In other words, the scalp EEG recording are ***V***_rec_ = ***V***_sim_ + Σ with size *n*×500, which is the input of the following reference techniques. Obviously, the reference of this simulated data ***V***_sim_ is at infinity.

### The EEG references

The potentials referenced to the cephalic (FCz), the occipital (Oz), the mean mastoids (MM) and the average (AVE) references, indicated by ***V***_FCz_, ***V***_Oz_, ***V***_MM_, and ***V***_AVE_ respectively, have been derived from the simulated EEG ***V***_rec_ according to the appropriate linear transformation (Yao, 2001). In particular, the MM reference signal has been modeled by the average of the TP9 and TP10 electrodes that are located in the proximity of the mastoids. The simulated EEG recording has been transformed according to the REST method (Yao, 2001), and a reconstruction of the infinity reference potential ***V***_REST_ was derived from ***V***_AVE_ based on the free software of REST (http://www.neuro.uestc.edu.cn/rest).

### Relative error for evaluation

The re-referenced potentials V^*^ were compared to the theoretical potential ***V***_sim_ to assess the effects of different references, and V^*^ was an alternative denotation of the recording ***V***_FCz_, ***V***_Oz_, ***V***_MM_, ***V***_AVE_ as well as ***V***_REST_. The degree of similarity between the re-referenced potentials and the theoretical simulation potential has been assessed by calculating the relative error (RE) according to the formula:

(5) $$\begin{array}{r} \text{RE }=\;||V_{\text{sim}}-V^{*}||/||V_{\text{sim}}|| \end{array}$$

where ||·|| denotes the matrix Frobenius norm. As the transformations of references are linear operations, we only simulated the performance according to the potential of a single vertex in each simulation, and the performance according to various dipole combinations can be deduced according to the linearity of the operation. We conducted 128 realizations with different the random seeds of the Gaussian white noise Σ for each dipole, in each signal-to-noise ratio and each number of electrodes. And the RE was averaged in these 128 realizations to obtain the performance of a single vertex. Then all the dipoles belonging to a large-scale brain network were averaged to obtain the performance of a single network.

## Results

### Signal strength and the sensitive/neutral electrode

**Figure 2:**
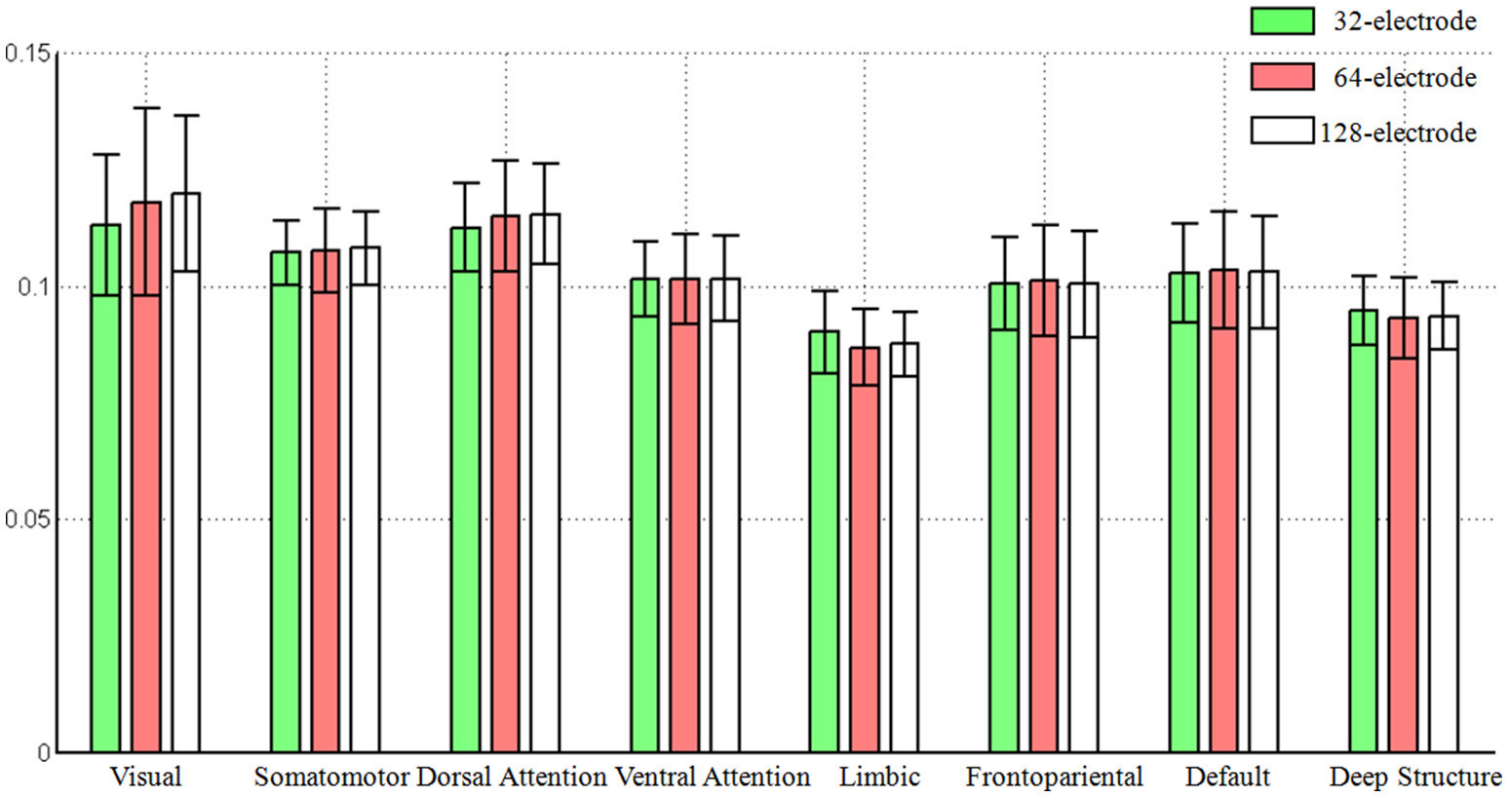
**The global sensitivity (averaged Frobenius norm) of large-scale network that can be observed in the scalp EEG**. The Frobenius norm of vertices were averaged across each large-scale brain network and the standard error was also calculated. The number of electrodes included 32, 64, and 128.

Based on the averaged Frobenius norm of the lead-field matrix, we obtained the global sensitivity of each large-scale network for each electrode configuration. We found the increased number of electrodes did not mean the increasing of the global sensitivity of a large-scale network that can be observed from the scalp surface, as the averaged norm was consistent with the same level (see Figure 2). Another interesting pattern was that visual network always had the largest sensitivity (0.1171, averaged for all electrode configuration), while limbic system had the smallest (0.0882).

**Figure 3:**
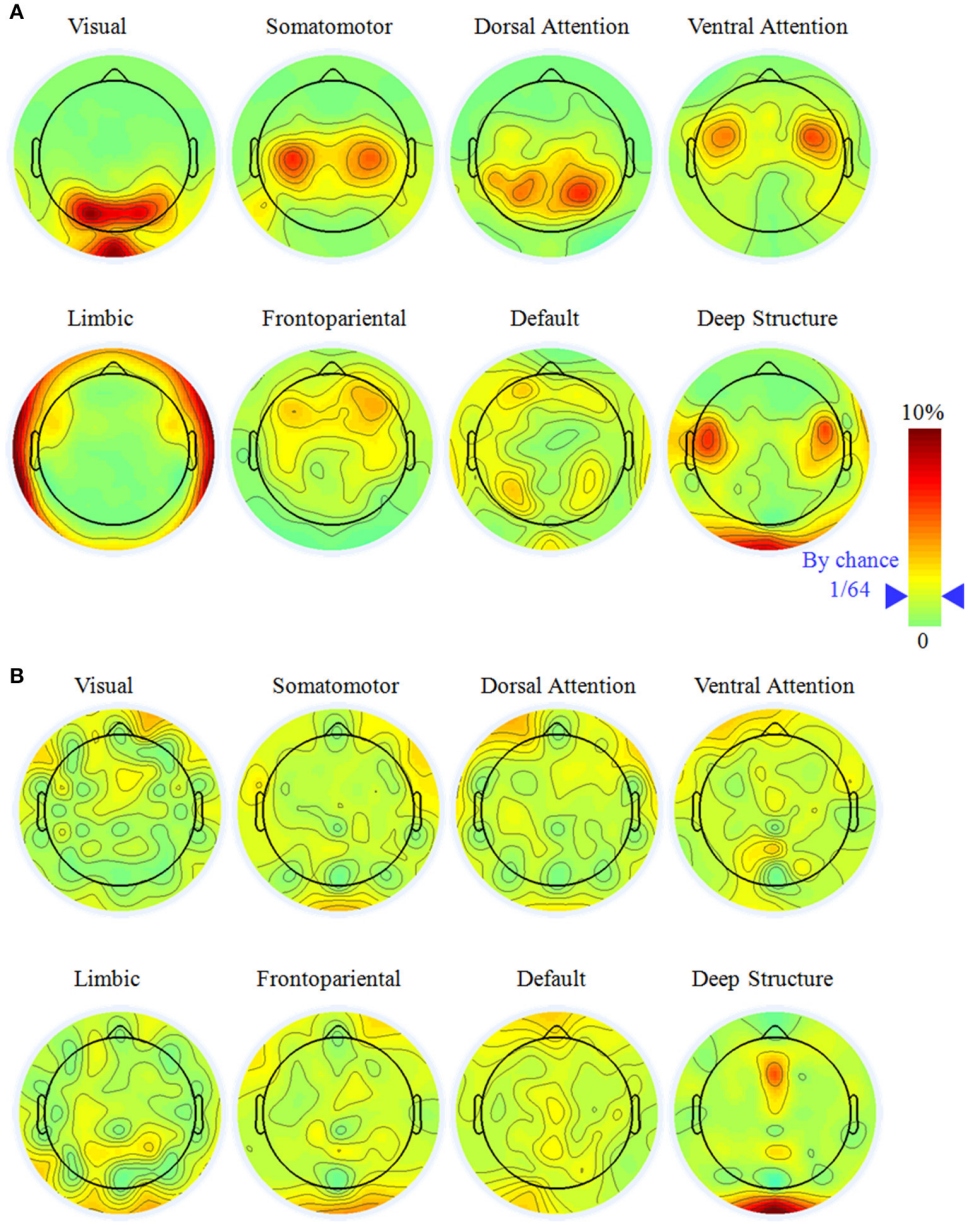
**The most sensitive (A)** and neutral electrodes **(B)** for each large-scale brain network with the electrode number of 64. Notice it would be 1/64 (marked in the color bar) if all the electrodes have equal chance to be the most sensitive or neutral electrode. The most sensitive electrode had a symmetric distribution and concentrated on specific electrode while the most neutral electrode was random.

For widely employed 64-electrode, we further considered the sensitivity to observe the large-scale networks in electrode level. Figure 3 illustrated the percentage topography of each electrode that was selected as the most sensitive or neutral electrode. Notice it would be 1/64 (marked in the legend of Figure 3) if each electrode had the same chance to be the most sensitive or neutral electrode. For the most sensitive electrode, the topography had a left-right symmetric pattern and the largest percentage electrodes neighbored to the superficial regions of a network. This phenomenon was apparent for the visual (PO3, PO4, and POz), the somatomotor (C3 and C4), the dorsal attention (P3 and P4), the ventral attention (FC3 and FC4), the limbic (FT9, FT10, TP9, and TP10) networks and the deep brain structure (FC5 and FC6). The above listed electrodes had percentage larger than 5%, which was triple of the random chance. We did not find any specific sensitive electrode for the frontopariental or the default network, as neither had any percentage of electrode larger than 4.2%.

It was hard to find the neutral electrode in the scalp surface, except in the deep brain structure. For the visual, somatomotor, dorsal attention, ventral attention, limbic, frontoparietal, and default networks, all the electrodes had the chance to be selected as the most neutral electrode with percentage less than 3.5%. For the deep brain structure, Oz and FCz had percentage of 6.8% and 6.1%, respectively, implying they were potential good reference for the study of the deep brain structure.

**Figure 4:**
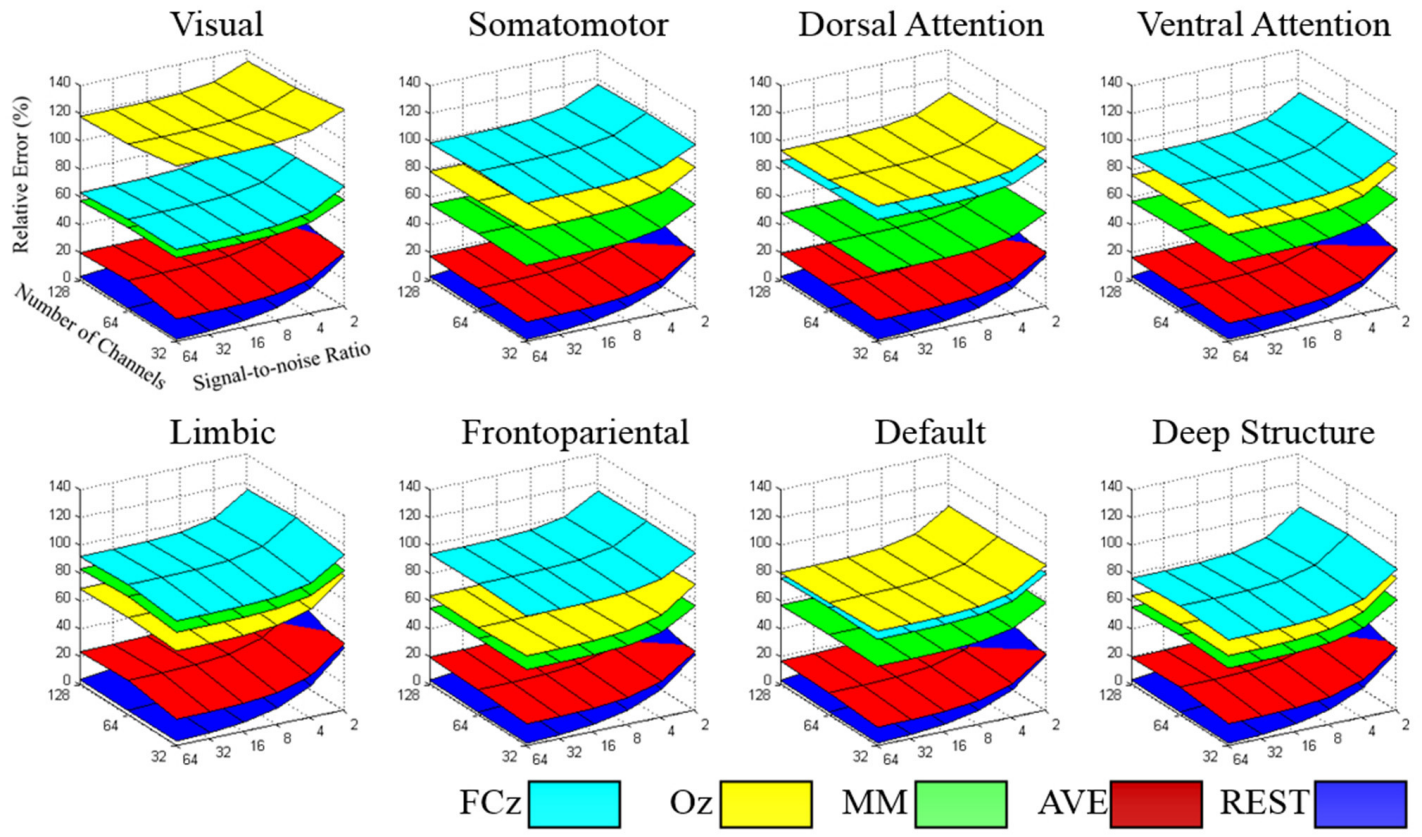
**The relative error of the re-reference signal to the theoretical true signal was calculated in each signal-to-noise level and electrode configuration, for each large-scale network**. The value of the relative error followed the pattern REST<AVE<MM<(FCz, Oz), which each network had its specific characteristic in reference selection.

The relative error of the re-reference signal to the theoretical true signal was calculated in each signal-to-noise ratio and electrode configuration, for each large-scale network. As illustrated in Figure 4, the relative error followed the pattern REST<AVE<MM<(FCz, Oz), as the number of electrodes and the signal-to-noise ratio constituted the coordinates (see Table 1 for an example dataset of 64-electrode). REST always had the best performance compared with any other reference system. For example, its relative error was less than 3% in the signal-to-noise ratio of 32 in 64-electrode (the fifth row of Table 1). However, the other references all had relative errors larger than 15% (the first to fourth rows of Table 1). For the dataset of 64-electrode, we conducted a 2-factor analysis of variance (ANOVA) in each large-scale network, with factors of signal-to-noise ratio (6) and reference electrode (5). All large-scale networks had significant main effect of reference electrode (*p* < 0.0001), and *F* values ranged from 342.51 (the deep brain structure) to 2,106.78 (the somatomotor network).

**Table 1:** **The relative error of the re-reference signal to the theoretical true signal was calculated in each signal-to-noise level, for each large-scale network, with the number of electrodes of 64**.

**SNR**	**Reference**	**The averaged relative error in each large-scale network, mean** ± **SE (%)**							
		**Visual**	**Somato-motor**	**Dorsal attention**	**Ventral attention**	**Limbic**	**Fronto pariental**	**Default**	**Deep structure**
64	FCz	65.4 ± 1.2	99.8 ± 1.5	86.4 ± 1.7	90.0 ± 2.1	93.1 ± 2.1	93.7 ± 1.9	76.7 ± 1.3	77.9 ± 2.7
	Oz	119.2 ± 2.3	78.5 ± 1.2	93.4 ± 1.8	75.6 ± 1.6	69.5 ± 1.9	62.5 ± 1.4	80.2 ± 1.4	64.7 ± 2.7
	MM	59.4 ± 1.1	55.1 ± 0.9	48.8 ± 1.0	57.0 ± 1.3	84.7 ± 2.2	54.5 ± 1.1	57.3 ± 1.1	57.0 ± 2.2
	AVE	21.6 ± 0.4	16.0 ± 0.3	18.0 ± 0.3	15.2 ± 0.4	24.6 ± 0.7	17.9 ± 0.4	15.7 ± 0.3	19.8 ± 0.7
	REST	1.5 ± 0.0	1.6 ± 0.0	1.4 ± 0.0	1.6 ± 0.0	2.9 ± 0.1	1.7 ± 0.0	1.8 ± 0.0	1.8 ± 0.0
32	FCz	65.6 ± 1.2	99.9 ± 1.5	86.5 ± 1.7	90.1 ± 2.1	93.2 ± 2.1	93.8 ± 1.9	76.8 ± 1.3	78.1 ± 2.7
	Oz	119.3 ± 2.3	78.7 ± 1.2	93.5 ± 1.8	75.7 ± 1.6	69.7 ± 1.9	62.7 ± 1.4	80.3 ± 1.4	65.0 ± 2.7
	MM	59.5 ± 1.1	55.2 ± 0.9	48.9 ± 1.0	57.1 ± 1.3	84.8 ± 2.2	54.6 ± 1.1	57.4 ± 1.1	57.2 ± 2.2
	AVE	21.8 ± 0.4	16.2 ± 0.3	18.1 ± 0.3	15.4 ± 0.4	24.9 ± 0.7	18.1 ± 0.4	15.9 ± 0.3	20.1 ± 0.7
	REST	2.8 ± 0.0	3.0 ± 0.0	2.8 ± 0.0	3.1 ± 0.0	4.3 ± 0.0	3.2 ± 0.0	3.2 ± 0.0	3.4 ± 0.0
16	FCz	66.0 ± 1.2	100.2 ± 1.5	86.8 ± 1.7	90.5 ± 2.1	93.6 ± 2.1	94.2 ± 1.9	77.3 ± 1.3	78.7 ± 2.7
	Oz	119.6 ± 2.3	79.1 ± 1.1	93.9 ± 1.8	76.3 ± 1.6	70.4 ± 1.9	63.3 ± 1.3	80.8 ± 1.4	65.8 ± 2.7
	MM	59.8 ± 1.1	55.6 ± 0.9	49.3 ± 1.0	57.6 ± 1.3	85.2 ± 2.2	55.1 ± 1.0	57.9 ± 1.0	57.8 ± 2.2
	AVE	22.3 ± 0.3	16.9 ± 0.2	18.7 ± 0.3	16.3 ± 0.4	25.6 ± 0.7	18.8 ± 0.4	16.8 ± 0.3	20.9 ± 0.7
	REST	5.5 ± 0.0	5.8 ± 0.0	5.5 ± 0.0	6.2 ± 0.0	7.6 ± 0.0	6.3 ± 0.0	6.2 ± 0.0	6.8 ± 0.0
8	FCz	67.3 ± 1.2	101.3 ± 1.5	87.9 ± 1.7	91.9 ± 2.1	95.1 ± 2.0	95.6 ± 1.9	78.8 ± 1.3	80.7 ± 2.6
	Oz	120.6 ± 2.2	80.4 ± 1.1	95.0 ± 1.7	77.9 ± 1.6	72.6 ± 1.8	65.3 ± 1.3	82.4 ± 1.3	68.5 ± 2.6
	MM	60.9 ± 1.1	56.9 ± 0.9	50.5 ± 1.0	59.1 ± 1.3	86.6 ± 2.1	56.5 ± 1.0	59.4 ± 1.0	59.8 ± 2.1
	AVE	23.9 ± 0.3	19.1 ± 0.2	20.5 ± 0.3	18.8 ± 0.3	27.9 ± 0.7	21.1 ± 0.3	19.2 ± 0.3	23.4 ± 0.7
	REST	10.8 ± 0.1	11.6 ± 0.0	10.9 ± 0.0	12.3 ± 0.0	14.6 ± 0.1	12.4 ± 0.0	12.2 ± 0.0	13.4 ± 0.1
4	FCz	71.5 ± 1.2	104.9 ± 1.4	91.4 ± 1.6	96.4 ± 2.0	100.0 ± 1.9	99.9 ± 1.8	83.7 ± 1.2	86.6 ± 2.5
	Oz	123.8 ± 2.2	84.8 ± 1.0	98.6 ± 1.6	83.0 ± 1.5	79.3 ± 1.6	71.4 ± 1.2	87.4 ± 1.3	75.8 ± 2.4
	MM	64.3 ± 1.1	61.1 ± 0.9	54.5 ± 0.9	63.7 ± 1.2	91.0 ± 2.0	61.1 ± 0.9	64.0 ± 1.0	65.6 ± 2.0
	AVE	28.5 ± 0.3	25.1 ± 0.2	25.6 ± 0.2	25.5 ± 0.3	34.6 ± 0.6	27.4 ± 0.3	25.8 ± 0.2	30.3 ± 0.6
	REST	21.6 ± 0.1	23.2 ± 0.1	21.8 ± 0.1	24.6 ± 0.1	28.9 ± 0.1	24.8 ± 0.1	24.4 ± 0.1	26.9 ± 0.1
2	FCz	84.2 ± 1.1	116.1 ± 1.3	102.5 ± 1.5	109.9 ± 1.8	115.8 ± 1.7	113.1 ± 1.7	98.1 ± 1.1	103.4 ± 2.2
	Oz	133.7 ± 2.0	97.9 ± 0.9	109.5 ± 1.5	97.9 ± 1.2	98.8 ± 1.4	88.3 ± 1.0	102.0 ± 1.1	95.1 ± 2.0
	MM	74.8 ± 1.0	73.4 ± 0.8	66.3 ± 0.8	77.1 ± 1.0	104.5 ± 1.8	74.7 ± 0.8	77.3 ± 0.9	81.2 ± 1.7
	AVE	41.1 ± 0.3	40.1 ± 0.1	39.1 ± 0.2	41.8 ± 0.2	52.2 ± 0.5	43.3 ± 0.2	41.7 ± 0.2	47.4 ± 0.5
	REST	43.3 ± 0.2	46.4 ± 0.1	43.6 ± 0.1	49.2 ± 0.2	57.7 ± 0.2	49.6 ± 0.2	48.7 ± 0.1	53.7 ± 0.3

A strong contender is AVE, which had much closer performance to REST, especially for small signal-to-noise ratio and high density of electrode. In the worst condition of signal-to-noise ratio of 2, REST decreased its performance greatly with the relative error larger than 40% in 64-electrode (the last bold row of Table 1). When comparing with AVE in signal-to-noise ratio of 2, though REST (39.11%) was better than AVE (41.18%) in 32-electrode, it became worse when density increased to 64-electrode (REST: 49.02% vs. AVE 43.3) and even twice the relative error in 128-electrode (REST: 81.38% vs. AVE 43.31%). Our statistical analysis in each brain network with signal-to-noise ratio of 2 and the number of electrodes of 64 further confirmed the poor performance of REST, as all the paired *t*-tests between REST and AVE had *p* < 0.0001 and the *t* value were ranged from −55.43 (the somatomotor network) to −11.49 (the visual network).

Though each reference had relative constant performance for different number of electrodes and signal-to-noise ratio, we found each brain network had its distinct pattern in the relative error. In the worst condition with the number of electrodes of 32 and signal-to-noise ratio of 2, the relative error was ranged from 100.7% (deep brain structure) to 140.5% (visual), implying the importance of reference selection. Intriguingly, when comparing the reference of FCz and Oz, FCz was better in the visual, dorsal attention and default networks, while Oz was better in the somatomotor, ventral attention, limbic, frontoparietal networks and the deep brain structure.

## Discussion

In current study, the effect of reference was examined under a common network parcellation of the human brain function containing eight large-scale networks. Based on the lead-field matrix, we found the distribution of the most sensitive electrode had a symmetric pattern, and each network preferred some specific electrodes. In contrast, the electrodes in scalp surface had approximately equal chance to be the most neutral electrode. We focused our simulations on some reference systems, including FCz, Oz, MM, AVE, and REST. The results showed that the magnitude of relative error followed the pattern of REST<AVE<MM<(FCz, Oz), regardless of the number of electrodes and the signal-to-noise ratio. Our findings suggested that REST was the most outstanding reference for all large-scale networks and AVE had much closer performance to REST than any other references. As ERPs and EEG experiments within the same behavioral domain always concern certain components relating to specific brain networks, our systems neuroscience comparisons revealed here may provide a valuable recommendation about reference selection for clinical and basic researches.

### Sensitive/neutral electrode for large-scale network

For the widely adopted density of 64-electrode, we investigated the probability of each electrode that can be selected as the most sensitive or neutral electrode. In fact, the lead-field matrix provided a pure measure of activity that a dipole with a unit strength can be represented with electrode in the scalp surface and the probability would be 1/64 if each electrode had equal chance to be selected. For the most sensitive electrode, the topography had a hemisphere symmetric pattern and the largest probability electrodes neighbored to the superficial regions of a network. These electrodes provided a potential observing window for experimental studies concentrating on a specific brain network.

Based on the minimum absolute value of the lead-field matrix, we proposed a quantitative measure for the relatively neutral or “quiet” reference location. Previous studies had mentioned that the neutral reference location did not exist anywhere on the body (Kayser and Tenke, 2010; Nunez, 2010), it seemed to be true as all the electrodes for seven large-scale networks have the percentage less than 3.5%. One exception is the deep brain structure, which has the highest probability of neutral electrodes around OIz and FCz.

The electrodes near the parietal-occipital junction (PO3, PO4, and POz) had the high probability to be the most sensitive electrode for the visual network. For example, visual evoked potential such as P100 component, is observed over the electrode around parietal-occipital region (Hillyard and Anllo-Vento, 1998). In classical experiment paradigm of spatial selective attention, attention to the stimulus location increased amplitude of the P100. Another widely utilized paradigm concentrated on the visual network is steady state visually evoked potentials (SSVEP), which are signals that are responses to visual stimulation at specific frequencies ranging from 3.5 to 75 Hz (Herrmann, 2001). The electrode around parietal-occipital junction would record the electrical activity at the same frequency of the visual stimuli.

The electrodes near the central (C3 and C4) had the high probability to be the most sensitive electrode for the somatomotor network (Figure 3A). In the brain-computer interface based on motor imagery, C3 and C4 are utilized to record the Mu and Beta rhythms during motor imaging (Lei et al., 2009). They were proved to have strong signal as overlaying the sensorimotor areas. Another potential concentrated on the somatomotor network is the lateralized readiness potential (LRP), which is considered as the representation of the activation of response-related processes, starting after response hand selection and at the beginning of motor programming (Coles, 1989). LRP are extracted from an array of electrodes located over central and neighboring areas.

The electrodes near the parietal lobe (P3 and P4) had the high probability to be the most sensitive electrodes for the dorsal attention network. These dorsal parietal electrodes are close to the intra parietal sulcus and superior parietal lobe, which are the basic structures of the dorsal attention network (Yeo et al., 2011). In the goal-directed attention paradigm, P300 recorded during effortful attention correlated significantly with parietal, effortful processes under the subject's active control (Ford et al., 1994). FC3 and FC4 had the high probability to be the most sensitive electrode for the ventral attention network. We should emphasize that the ventral attention network is an aggregate of multiple networks such as the salience and the cingulo-opercular networks in the literature (Yeo et al., 2011).

### Choice of reference for large-scale network

We had a systematic simulation for the reference of FCz, Oz, the mean mastoids, the average and the infinity reference, with the number of electrodes from 32 to 128 and signal-to-noise level from 2 to 32. Our simulations demonstrated that the off-line re-reference techniques has distinct performance for different large-scale network, and always followed the pattern REST<AVE<MM<(FCz, Oz). In addition, the infinity reference performed by REST can substantially reduce the relative error.

Because FCz and Oz are always an electrically sensitive positions, there is some consensus in the literature that all cephalic reference such as FCz and Oz may be not a preferential choice for the measurement of local activity at cephalic target position (Hagemann et al., 2001). We found both FCz and Oz references may substantially distort the EEG potentials. In worst condition with 32-electrode and the signal-to-noise ratio of 2, both references had relative error larger than 100% in some networks. As FCz and Oz were close to frontal eye field and occipital regions respectively, the large-scale networks around these regions had been affected. We found FCz was better in visual, dorsal attention, default, while Oz was better in somatomotor, ventral attention, limbic, frontoparietal, and deep brain structure. In simultaneous EEG-fMRI study, FCz was frequently served as online reference in a nonmagnetic MRI-compatible EEG system (Lei et al., 2014). This may because it is easy to be affixed to the surface of the scalp and it has typical MRI imaging artifact, and the latter is crucial in off-line process to remove gradient and ballistocardiographic (BCG) artifacts.

Although it has been argued that the mastoids are relatively inactive, this has been persuasively shown to be false (Hagemann et al., 2001). In our simulation, the mean mastoids (MM) was always better than FCz and Oz because the average of bilateral mastoids regions was substantially less active for most networks. However, there was an exception for the limbic network, which followed the pattern Oz<MM<FCz in relative error. In the condition of signal-to-noise ratio of 64 and number of electrodes of 64, the relative errors of MM was 84.7% while that of Oz was 69.5% in the limbic network.

The average reference is commonly recommended reference, and it assumed that the average across all scalp electrodes at each time point is substantially less active than the target sites. Theoretically, if the head is assumed to be a concentric sphere structure with homogenous conductivities within each sphere, then an ensemble of dipoles inside the sphere would generate an electric field such that the integral of the potential on the surface of the sphere is zero at any given time point (Bertrand et al., 1985). This is valid only with sufficient electrode density and full coverage of the head surface (Yao, 2017). Otherwise the AVE reference was not completely free of biases as the spatial sampling being limited to the upper part of the head (Nunez and Srinivasan, 2006). Our simulation proved that the AVE reference was quite a neutral reference, and had the best performance when compared with FCz, Oz, or MM. More importantly, the performance of AVE can even be better than REST for high density electrode, and we will back to this point hereinafter.

With the application of high density electrode and modern computation technique, a better reference, named infinity reference, was developed based on a reference electrode standardization technique (REST) (Yao, 2001). As it approximately reconstructed a point far away from all the scalp electrodes, REST provided a neutral reference. Previous studies of simulation and experiments showed that REST is very effective for most important superficial cortical region. We specified this simulation to the whole brain, with the common network parcellation of the human brain function, offering a system perspective of the performance of REST. Previous studies have shown the availability of high-density EEG systems and an accurate knowledge of the head model are crucial elements to improve REST performance (Zhai and Yao, 2004; Liu et al., 2015; Chella et al., 2016).

In this work, a particular emphasis has been placed on the comparison between the REST and AVE references, the superiority of one method over the other having been the subject of some debates (Kayser and Tenke, 2010; Nunez, 2010). Based on the findings of our simulation, we concluded that REST can provide superior performance than AVE in reducing the reference bias even when the signal-to-noise ratio decreased to 4. However, when noise was extremely large, or the electrodes were high-density, AVE may be a strong rival to REST. This is in line with previous finding that REST was sensitive to noise and the number of electrodes (Liu et al., 2015). Though REST (38.3%) was better than AVE (40.3%) when the number of electrodes was 32, it was worse when electrode is increase to 64 (REST: 47.8% vs. AVE 42.1%) and 128 (REST: 79.4% vs. AVE 42.2%). This is essentially due to REST assuming the sources of the EEG recordings lying inside the equivalent source distribution (ESD). Since the instrumental noise is not generated by sources inside the ESD, the effectiveness of the standardization to a reference point at infinity becomes less accurate in comparison to the noiseless case (Zhai and Yao, 2004).

### EEG reference under the perspective of large-scale network

The choice of reference has substantial effects on analysis and interpretation, and the optimal choice of reference site depended on the study domain and purpose of the analysis. In our current study, we considered a special application scenario: behavioral paradigm focused on some special large-scale brain networks. This is rational for most model-driving experiments because brain regions are always included in the assumptions before the EEG or ERPs studies. Both the evoked and the spontaneous potentials of neural activities are currently interpreted in terms of components generated by distinct large-scale network. Because of the complex influence of the spatiotemporal characteristics of skull volume conduction on the EEG signal, it is still hard to estimate the related large-scale networks from the signal in scalp surface (Lei et al., 2011). However, the application of the high-density EEG, especially the wide application of fMRI, makes it acceptable and even normal to include some assumptions about the localization of source before an EEG or ERPs study.

**Figure 5:**
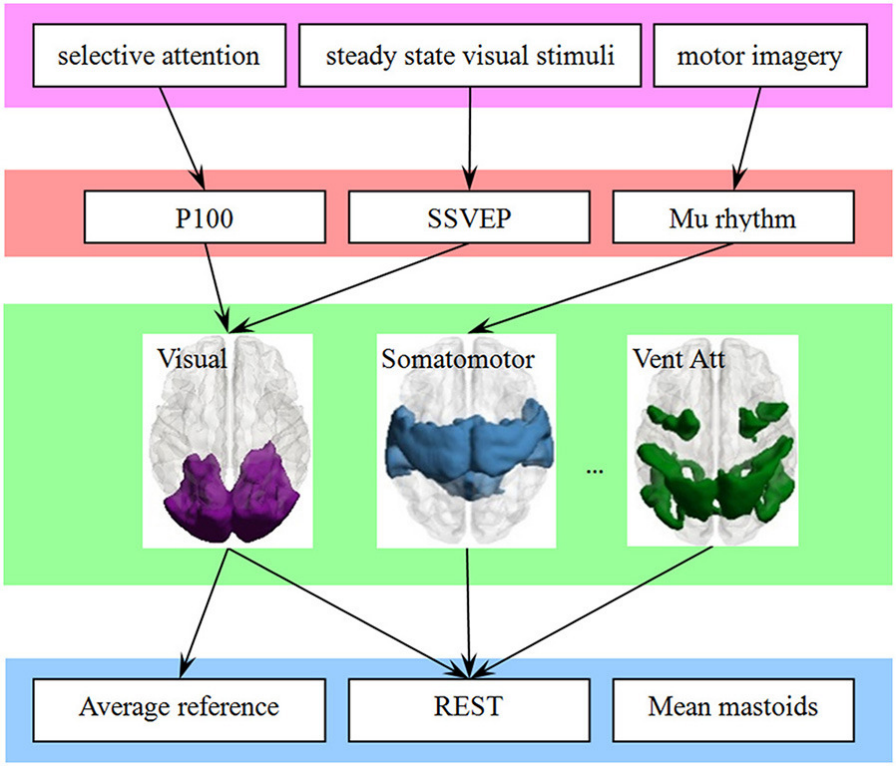
**The large-scale brain network is a bridge to link the behavioral paradigm to the EEG studies, which may simplify the choice of reference**. A behavioral paradigm (first row) often engages several EEG or ERPs components (second row) that are in turn supported by multiple brain regions, which constructed a large-scale network (third row). In addition, each large-scale network has its specific sensitive or neutral electrodes. The choice of electrode is simplified if the link from electrophysiological components (fourth row) to large-scale network is finite and consistent.

The large-scale brain networks, which are constructed from the intrinsic spontaneous activity of resting-state function MRI, were found have a constant spatial pattern in task conditions (Smith et al., 2009; Yeo et al., 2015). As illustrated in Figure 5, we thought the large-scale brain network might be a bridge to link EEG and ERP components in one hand, and the choice of EEG reference in another hand. Previous neuroimaging studies have revealed that each network is functional specialization, and we thought this will lead to a many-to-one mapping between electrophysiological components and the large-scale network.

The attribution of functional specialization illustrated in Figure 5 may be considered at different spatial scales (Gilbert et al., 2010). For example, at the macro-scale, the visual network can be described as being specialized for visual processing, and the reference scheme of visual ERPs such as P100 or N170 may be inferred at this macro-scale. At a finer scale, sub-regions of the visual network may be distinguished based on their sensitivity to different visual features. A fine-resolution parcellation of the cerebral cortex can increase the accuracy of the choice of electrode. For example, N170 may indicate its reference scheme from the sub-network around the lateral visual areas. The template we utilized also has a fine-resolution with 17-network parcellation of the human cerebral cortex based on 1,000 subjects (Yeo et al., 2011). This detailed parcellation corresponded to a hierarchical behavioral domain classification (Poldrack and Yarkoni, 2016), and may lead to a more relevant reference selection.

Our current simulations have several limitations, especially separating a vertex to a specific large-scale network. In fact, the same vertex can belong to different networks. In addition, if all or majority of vertices in a network are active simultaneously, the most sensitivity\neutral electrodes and the relative error of each re-reference method would be extremely different from the current results. More important, it is in this way that the results can be interpreted in terms of what happens when a particular network is functioning. And experiments may find a guide on which reference and what type of analysis are adequate when the activity of interest is related with/generated from a particular brain network.

## Summary and conclusion

The choice of an EEG reference is an important initial step for EEG analysis, and the findings of different reference schemes should be treated as interchangeable (Hagemann et al., 2001). Our findings suggested that REST was a potential reference for all the large-scale networks and AVE was much closer in performance to REST. This large-scale network approach, based on a large sample of published neuroimaging studies, can reassign large bodies of EEG and ERPs signal of distinct tasks with novel organizational features at the systems level, thereby offering potential reference electrode for clinical studies and research studies using EEG and ERPs.

## Author contributions

Conceived and designed the experiments, performed the experiments and analyzed the data: XL. Contributed reagents/materials/analysis tools: XL and KL.

### Conflict of interest statement

The authors declare that the research was conducted in the absence of any commercial or financial relationships that could be construed as a potential conflict of interest.
